# Contribution and effectiveness of ski and pole forces in selected roller skiing techniques on treadmill at moderate inclines

**DOI:** 10.3389/fspor.2023.948919

**Published:** 2023-02-22

**Authors:** Shuang Zhao, Stefan Lindinger, Olli Ohtonen, Vesa Linnamo

**Affiliations:** ^1^ Faculty of Sport and Health Sciences, University of Jyväskylä, Jyväskylä, Finland; ^2^ Center of Health and Performance (CHP), Department of Food and Nutrition and Sport Science, University of Gothenburg, Gothenburg, Sweden

**Keywords:** double poling technique, gear 3 technique, speed maintain, crosscountry skiing, effectiveness

## Abstract

**Background:**

Most of the studies about the effects of incline on cross-country skiing are related to the metabolic efficiency. The effective skiing biomechanics has also been indicated to be among the key factors that may promote good performance. The aims of this study were to provide biomechanical characteristics and investigate the relative contribution and effectiveness of ski and pole forces in overcoming the total external resistance with double poling (DP) and Gear 3 (G3) techniques at varying moderate uphill inclines.

**Methods:**

10 male cross-country skiers participated in this study. Custom-made force measurement bindings, pole force sensors, and an 8-camera Vicon system were used to collect force data and ski and pole kinematics at 3°, 4° and 5° with 10 km/h skiing speed.

**Results:**

The cycle length (CL) decreased by 10% and 7% with DP and G3 technique from 3° to 5° (*p* < 0.001, *p* < 0.001). The cycle rate (CR) increased by 13% and 9% from 3° to 5° with DP and G3 technique respectively. From 3° to 5°, the peak pole force increased by 25% (*p* < 0.001) and 32% (*p* < 0.001) with DP and G3 technique. With DP technique, the average cycle propulsive force (ACPF) increased by 46% (*p* < 0.001) from 3° to 5°and with G3 technique, the enhancement for ACPF was 50% (*p* < 0.001). In G3 technique, around 85% was contributed by poles in each incline.

**Conclusion:**

The higher power output in overcoming the total resistance was required to ski at a greater incline. With DP technique, the upper body demands, and technical effectiveness were increasing with incline. With G3 technique, the role of external pole work for propulsion is crucial over different terrains while role of legs may stay more in supporting the body against gravity and repositioning body segments.

## 1. Introduction

Cross-country (XC) skiing is a sport in which competition and training are normally performed on varying track topography. The classical and skating style (also known as freestyle) are the two basic skiing techniques. Techniques such as double polling, diagonal stride, and kick double pole, are sub-techniques of classical skiing technique (1). In skating technique, there are six different sub-techniques, so called gears (Gear 2-Gear 7) (2, 3). In both classical and skate skiing, skiers change the sub-techniques spontaneously to maintain high speed and adapt to the change of the terrain (4–6). Several researchers have studied the effect of incline and speed on metabolic efficiency (7–9), technique shift (5, 10), as well as kinematics and kinetics change (11–16). The effects of incline on metabolic efficiency have been studied a lot (7–9), as it is a key factor for endurance sports (17). The effective skiing biomechanics has also been indicated to be among the key factors that may promote good performance (18). Therefore, having more knowledge about the effects of incline on skiing biomechanics may be beneficial for skiers and coaches with skiing technique improvements for maintaining the skiing speed at varying uphill inclines.

Several studies have investigated the effects of incline on cycle and force characteristics of different skiing techniques. The cycle rate (CR) has been proved to be higher at steeper inclines (4, 19). In both DP and Gear 2 (G2) technique, the peak pole force (PPF), average pole force and average cycle pole force were all greater at the higher incline situations (19). The primary mechanical determinant of skier's performance is the propulsive force (1), which has been defined as the forward directed component of the 3D resultant reaction force from skis and poles acting on skiers (1, 16, 20–22). The total external resistance should be overcome by the total propulsive force in XC skiing. However, less works (16, 22) have examined the effects of incline on the forces and propulsive forces generated from skis.

As one of the main techniques in classic XC skiing, the usage and the importance of DP technique have been increased during the past years due to increased upper body power, more systematic strength training and higher skiing speeds (20, 23). The Gear 3 (G3) technique has also become the most commonly used technique in the freestyle XC skiing competition (24). DP and G3 technique are normally used in level terrain up to moderate uphill inclines. The DP technique, which involves both arms acting in unison and leg involvement, has often been considered as an upper-body movement (25–27) as the propulsive forces are exerted only through the poles even though it is clear that also legs contribute to the performance (28, 29). The G3 contains symmetrical pole thrust on every leg stroke (1). The propulsive force in G3 are generated from both skis and poles (30, 31). Although most of the total propulsive force has been proved to be attributed to the forces from poles in skate skiing techniques (32, 33), how the ski and pole forces are performed to maintain the speed with varying uphill inclines need further investigation.

Therefore, the current study was conducted to (1) provide biomechanical characteristics and (2) investigate the relative contribution and effectiveness of ski and pole forces in overcoming the total external resistance of both DP and G3 techniques at varying moderate uphill inclines. We hypothesized that with DP technique, we could measure some propulsive forces from skis, but it would be quite small, and pole forces would be more effective at steeper inclines than at relative lower inclines. We also hypothesized that pole forces contribute more and would be more effective than the ski forces with G3 technique (32, 33) in overcoming the total resistance at any incline, but the relative contribution of ski forces to overcome the total resistance would increase at steeper grade.

## 2. Materials and methods

### 2.1. Participants

10 male participants (age: 29.4 ± 7.9 years; height:181.4 ± 5.7 cm; weight:77.9 ± 8.9 kg) who were familiar with treadmill roller-skiing volunteered to participate in this study. The participants' group included experienced skiers, such as high-level junior athletes, recently retired athletes from the national team, local skiing club members and national team coaches, both latter ones are with high roller skiing skill and fitness levels. All protocol used in this study were approved by the Ethics Committee of the University of Jyväskylä. All participants were provided written informed consent before the measurement and were free to withdraw from the experiments.

### 2.2. Protocol

Passive reflective markers were attached onto skiing equipment before the measurement. First, participants completed a 10–15 min warm-up roller skiing on the treadmill. After the warm-up activity, the DP technique was performed at 3°, 4° and 5° at a speed of 10 km/h. This speed is commonly used in aerobic capacity tests where the speed is kept constant. There was a 1-min rest between each incline. When the DP technique was done, pole length was adjusted to a comfortable length for G3 technique (1). The comfortable pole length for DP technique were 85.9% ± 2.5%, and for G3 were 90.0% ± 1.3% of skiers' body height in this study. The participants were given a 5-min rest period while adjusting the pole length. The G3 technique was then performed on the treadmill. The protocol for changing the incline was the same as during the DP test.

### 2.3. Data collection

**Figure 1:**
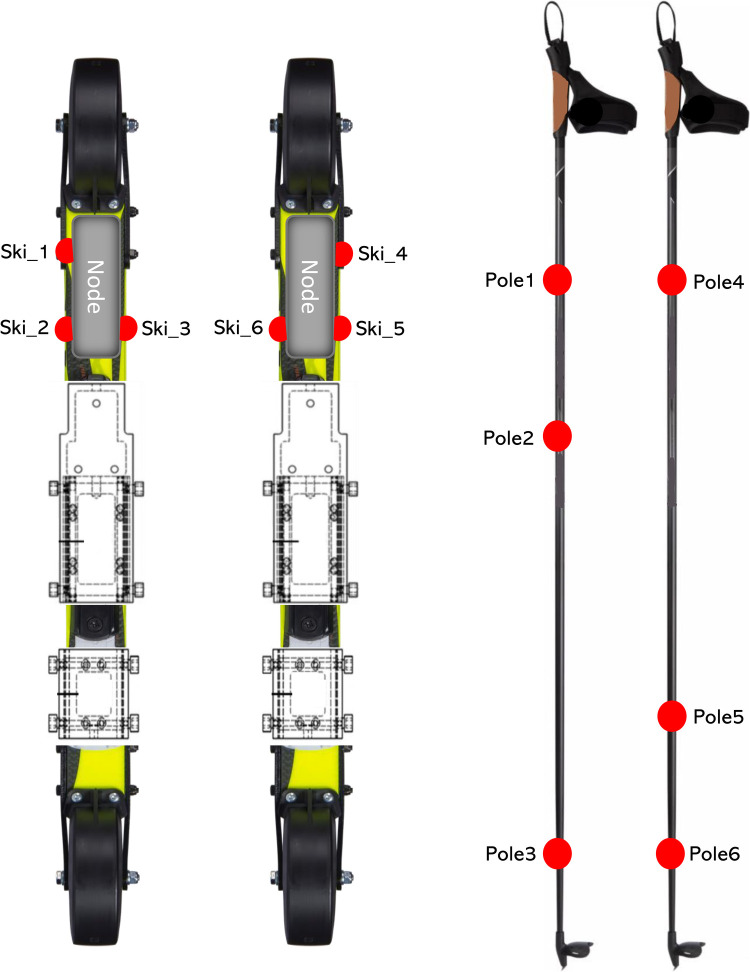
Illustration of the marker placement on skis and Poles.

An 8-video-camera motion capture system and NEXUS 2.8.1 software (Vicon, Oxford, United Kingdom) were used to collect and record the three-dimensional (3D) trajectories of reflective markers at a sampling rate of 150 Hz. The global coordinate system (GCS) was defined by using the right-hand rule when the incline of the treadmill was 0° and was calibrated according to Vicon's specifications. The Y-axis of GCS was defined as the longitudinal axis of the treadmill. The Z-axis of GCS was perpendicular to the ground pointing upward. 15 reflective markers were used in this study. 6 markers were attached onto the roller skis (3 markers each, Figure 1) and 6 markers were attached onto the poles (3 markers each, Figure 1). Another 3 markers were attached onto the treadmill. Two markers were attached to the front and rear right corners of the treadmill. Another one was attached to the rear left corner of the treadmill. All markers in this study were used to provide the position of roller skis, poles, and the treadmill in the GCS.

Measurements were performed on a motorized treadmill with a belt surface 2.7 m wide and 3.5 m long (Rodby Innovation AB, Vänge, Sweden). A same pair of roller-skis (Marwe SKATING 620 XC, wheel no. 0, Marwe Oy, Hyvinkää, Finland) were used for both techniques and all participants. Two custom-made pole force sensors (VTT MIKES, Technical Research Centre of Finland Ltd., Kajaani, Finland) were used to measure axial ground reaction force (GRF) from poles at a sample rate of 400 Hz. The pole force sensors were mounted below the pole grip and were calibrated in a certified laboratory for force and mass measurements (VTT MIKES, Technical Research Centre of Finland Ltd., Kajaani, Finland). Two custom-made 2D (vertical and medio-lateral) force measurement bindings (Neuromuscular Research Centre, University of Jyväskylä, Finland) (34) were mounted on the roller-skis to measure the leg forces generated from roller-skis at a sampling rate of 400 Hz. Both pole force sensor and ski measurement bindings have been used in our previous study (35). The total mass of one equipped pole and one equipped roller ski were 202 g and 664 g greater than the normal ones. A trigger signal was sent from the Coachtech online measurement and feedback system (36) (Neuromuscular Research Centre, University of Jyväskylä, Finland) to the motion capture system to mark the start of the force capture. Data from each subject at each incline were collected for at least 30s when the treadmill speed was constant at 10 km/h.

### 2.4. Data processing

**Figure 2:**
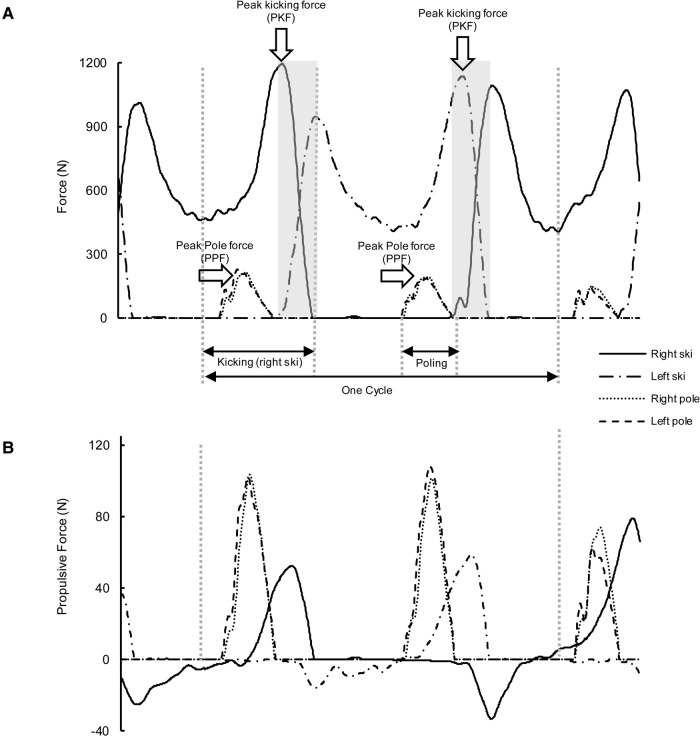
Definition of cycle and force variables and time-force curve of propulsive force with G3 technique. A. Definition of cycle and force variables. Areas under the gray shade were the overlap phase. B. Time-force curve of propulsive force from skis and Poles.

The marker labeling was performed by using NEXUS 2.8.1 software. The raw 3D trajectories of all reflective markers were low-pass filtered (fourth-order, zero-lag, Butterworth filter) with a cut-off frequency of 11.3 Hz (37). Force data were low-pass filtered (eighth-order, zero-lag, Butterworth filter) with a cutoff frequency of 15 Hz (38). Filtering and parameter calculation were performed in MATLAB R2018a (MathWorks, Natick, United States). 10 cycles from each DP technique trail and 5 cycles from each G3 technique trail were analyzed in this current study. For DP technique trails, one cycle was defined as the period between two consecutive right pole plant. For G3 technique, one cycle was defined as the time between consecutive same side ski force minima after ski plant and contained the kicking, overlapping, pure gliding action of both left and right ski and two double poling action from both poles (Figure 2A).

#### 2.4.1. Propulsive force calculation

Forces measured in the force coordinate system were first transformed into the GCS (35). The measured axial pole forces were considered as the ground reaction forces acting along the pole from the tip to the top. The pole forces vector ($\vec{{\;F}_{\;\;pole}}$) in GCS were calculated as:.

$$\vec{{\;F}_{\;\;pole}}=F*\;\vec{u}$$

where F was the magnitude of the measured axial pole force and $\;\vec{u}$ was the direction vector from the tip to the top of the pole. The direction vector was defined by using the reflective markers that were attached to the pole. As the measurements were performed at different inclines, the propulsive force component from ski (F_p_ski_) and from pole (F_p_pole_) were calculated by:

$$F_{{\;p}_{\_\mathrm{ski}}}=F_{\mathrm{skiy}}*\cos\alpha +F_{\mathrm{skiz}}*\sin\alpha$$

$$F_{{\;p}_{\_\;\mathrm{pole}}}=F_{\;\mathrm{pole}\_y}*\cos\alpha +F_{\;\mathrm{pole}\_z}*\sin\alpha$$

where *α* was the incline of the treadmill (3°, 4°, or 5°); F_pole_y_ and F_pole_z_ were the corresponding pole force components in GCS; F_skiy_ and F_skiz_ were the components of GRFs' vector generated from legs in GCS.

#### 2.4.2. Cycle characteristics

The cycle rate (CR) for each technique was the cycles per second (CR = 1/Cycle time, Hz). The cycle length (CL) was defined as the product of cycle time and the speed of the treadmill. In both techniques, the poling time was the ground contact time of the right poles. For G3 technique (Figure 2A), the kicking time of one leg was the time from unweighting minima to the ski release. The overlap time of the legs were defined as the time from one ski plant to the adjacent ski release of the other ski. The relative poling, kicking, and overlap times were calculated for the analysis.

#### 2.4.3. Impulses, effectiveness and contributions of ski and pole forces

The kinetic variables analyzed in this study were similar to those in another study which we concentrated on the effect of changing the treadmill speed. The peak pole force (PPF) for both techniques, and peak kicking force (PKF) for G3 technique were determined by the resultant force from pole and ski respectively (Figure 2A). For both techniques, pole and ski propulsive force impulses as well as ski vertical impulse were calculated. The propulsive force impulse was equal to the cumulative time integral of the propulsive force, restricting the integral to the intervals over which the integrand was positive. The effectiveness index of pole and ski forces was calculated by expressing the pole propulsive impulse and the ski propulsive impulse as a percentage of pole and ski resultant force impulse, respectively (16). The contribution of pole and ski forces in overcoming the total resistance were calculated by expressing the pole and ski propulsive impulse as a percentage of total propulsive impulse (32), respectively. The average cycle propulsive force (ACPF) were determined by dividing the total propulsive impulse by cycle time (16). The power output in overcoming the total resistance in skiing direction was calculated by multiplying the APCF and the speed of the treadmill (m/s) (16).

### 2.5. Statistical analyses

All the data in this current study were shown as means ± SD. One-way ANOVA with repeated-measures and *Bonferroni post hoc* analysis were conducted to reveal the effect of incline on each characteristic. The effect size ($\eta_{p}^{2}$) and statistical power were also provided for further evaluation. The level of statistical significance was set at 0.05. All statistical analyses were carried out by using SPSS 22.0 Software (SPSS Inc., Chicago, United States.).

## 3. Results

**Figure 3:**
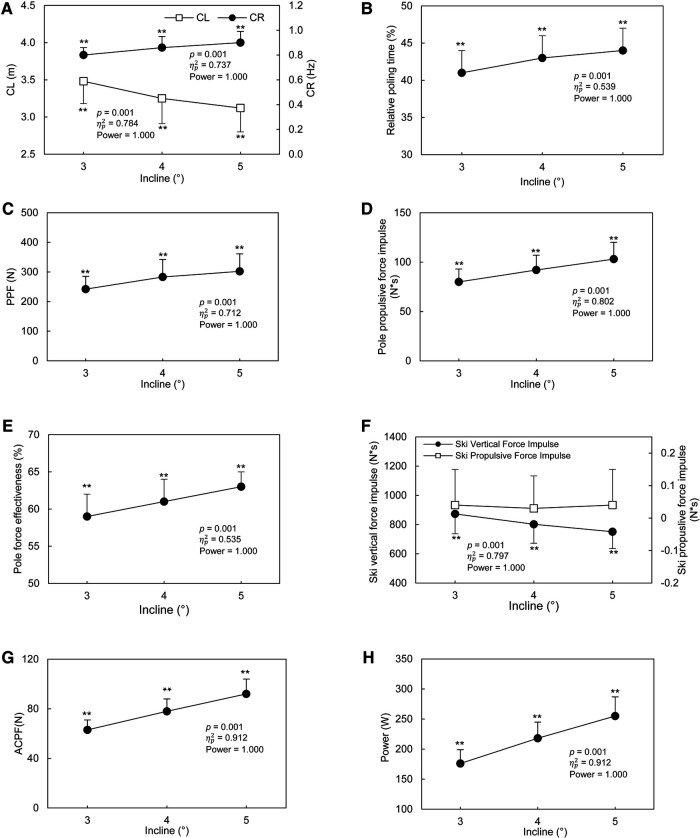
Cycle and kinetic characteristics of DP technique at different inclines. A: Cycle length (CL, left axis) and Cycle rate (CR, right axis); B: Relative poling time (%); C: Peak Pole force (PPF); D: Pole propulsive force impulse; E: Pole force effectiveness; F: Ski vertical force impulse (left axis) and Ski propulsive force impulse (right axis), G: Average cycle propulsive force (ACPF), H: Power output in overcoming the total resistance (Power). The data are presented as mean ± SD. The *p* value, $\eta_{p}^{2}$, and power presented in the figure are from the One-Way ANOVA with repeated measurement test. **p < 0.01, compared with all other inclines.

In DP technique, the CL (Figure 3A) decreased by 10% as the incline of the treadmill elevated from 3° to 5° (*p* < 0.001). The CR (Figure 3A) and relative poling time (Figure 3B) increased by 13% (*p* < 0.001) and 7% (*p* < 0.001) from 3° to 5°, respectively. From 3° to 5°, PPF increased by 25% (*p* < 0.001, Figure 3C) and the pole propulsive force impulse increased by 29% (*p* < 0.001, Figure 3D). The pole force effectiveness increased by 7% from 3° to 5° (*p* < 0.001, Figure 3E). With DP technique, the ski vertical force impulse decreased with the increasing incline (*p* < 0.001, Figure 3F). The ski propulsive force impulse was small and independent from the incline of the treadmill (*p* = 0.284, Figure 3F). The ACPF and the power output in overcoming the total resistance increased by 46% (*p* < 0.001, Figure 3G) and 45% (*p* < 0.001, Figure 3H) with DP technique, respectively.

**Figure 4:**
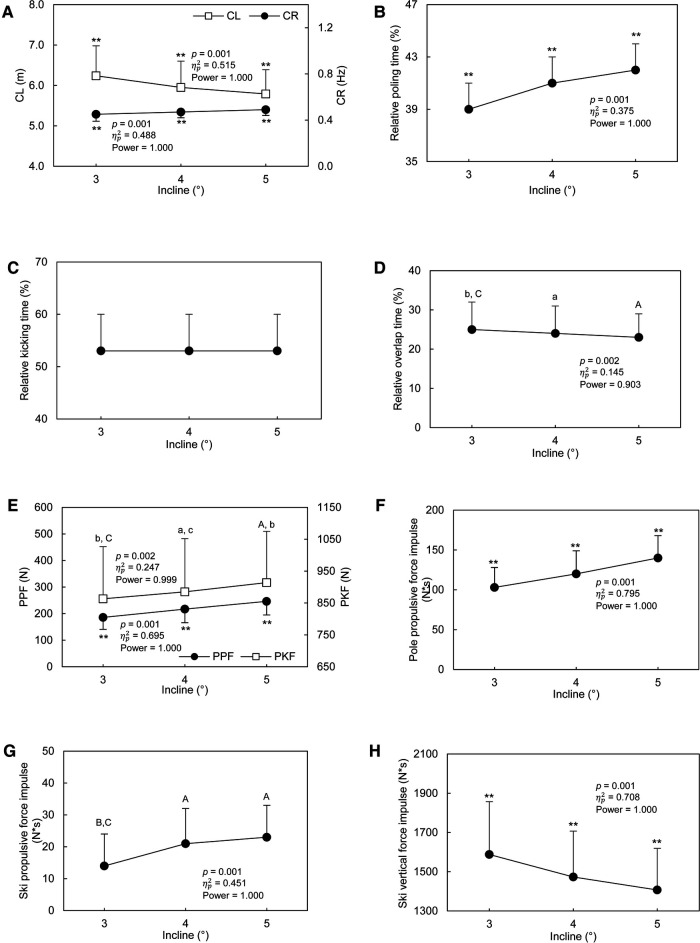
Cycle and kinetic characteristics of G3 technique at different inclines. A: Cycle length (CL, left axis) and Cycle rate (CR, right axis); B: Relative poling time (%); C: Relative kicking time (%); D: Relative overlap time (%); E: Peak Pole force (PPF, left axis), Peak kicking force (PKF, right axis); F: Pole propulsive force impulse; G: Ski propulsive force impulse; H: Ski vertical force impulse. The data are presented as mean ± SD. The *p* value, $\eta_{p}^{2}$, and power presented in the figure are from the One-Way ANOVA with repeated measurement test. **p < 0.01, compared with all other inclines. a, A; b, B; c, C, represent different to 3°, 4°, 5°, respectively. a, b, c = *p* < 0.05; A, B, C = *p* < 0.01.

**Figure 5:**
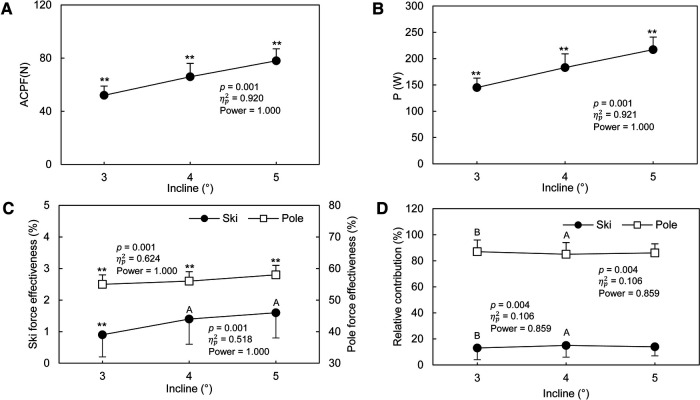
Kinetic characteristics of G3 technique at different inclines. A: Average cycle propulsive force (ACPF); B: Power output in overcoming the total resistance (Power); C: Ski force effectiveness (left axis) and Pole force effectiveness (right axis); D: Relative contribution of ski and pole forces in overcoming the total resistance. The data are presented as mean ± SD. The *p* value, $\eta_{p}^{2}$, and power presented in the figure are from the One-Way ANOVA with repeated measurement test. **p < 0.01, compared with all other inclines. a, A; b, B; c, C, represent different to 3°, 4°, 5°, respectively. a, b, c = *p* < 0.05; A, B, C = *p* < 0.01.

With G3 technique, the CL (Figure 4A) decreased by 7% as the incline of the treadmill elevated from 3° to 5° (*p* ≤ 0.001). The CR (Figure 4A) and the relative poling time (Figure 4B) with G3 technique increased by 9% (*p* < 0.001) and 8% (*p* ≤ 0.008) from 3° to 5°, respectively. With G3 technique, the relative kicking time (Figure 4C) was independent from the incline (*p* = 0.794). The relative overlap time (Figure 4D) at 3° was greater than relative overlap time at 4° and 5° (*p* = 0.101). From 3° to 5°, the PPF and the PKF increased by 32% (*p* < 0.001, Figure 4E) and 6% with G3 (*p* ≤ 0.037, Figure 4E) technique, respectively. From 3° to 5°, the pole propulsive force impulse increased by 36% (*p* < 0.001) with G3 technique (Figure 4F). The ski propulsive force impulse (Figure 4G) at 4° was not different from that at 5° (*p* = 0.338), but both were greater than the ski propulsive force impulse at 3° (*p* < 0.001). The ski vertical force impulse decreased by 11% from 3° to 5° (*p* < 0.001, Figure 4H). With G3 technique, the enhancements for ACPF were 50% (*p* < 0.001, Figure 5A). The power output in overcoming the total resistance increased by 50% (*p* < 0.001, Figure 5B). The pole force effectiveness (Figure 5C) increased by 5% (*p* < 0.001). The ski force effectiveness (Figure 5C) at 3° was significantly lower than at 4° to 5° (*p* < 0.001, *p* < 0.001). No significant difference on ski force effectiveness between 4° and 5° was found (*p* = 0.101). In G3 technique, around 85% of the total propulsive force was contributed by poles (Figure 5D). The relative contributions of ski and pole forces to overcome the total resistance were affected by the treadmill incline (Figure 5D), but the only difference was between 3° and 4° (*p* = 0.003).

## 4. Discussion

This study provided the biomechanical characteristics and investigated the relative contribution and effectiveness of ski and pole forces in overcoming the total external resistance of both DP and G3 techniques at varying moderate uphill inclines. 0.03–0.04 N*s ski propulsive force impulse was found, and the pole force effectiveness increased by 7% from 3° to 5°with DP technique, which support our hypothesis that some propulsive forces from skis could be measured but it would be quite small and pole forces would be more effective at steeper inclines. With G3 technique, 55%–58% of the resultant pole forces was generated to overcome the external resistance and about 85% of the total propulsive force was contributed by poles. Thus, the hypothesis of more contribution from poles and greater pole effectiveness was satisfied.

### 4.1. Cycle characteristics

In response to increases in incline, the CR increased significantly at steeper inclines with both techniques (Figures 3A, 4A). This indicated that shorter time was used by subjects to finish one cycle at steeper inclines. This finding is consistent with previous studies that the CR was higher at steeper inclines with G3 technique (4), and DP technique (19). Since the treadmill speed remained the same at different incline, the CL decreased at steeper inclines with both techniques. Similar finding was found in a previous study that the CL was decreased with both G2 and DP techniques in response to a steeper incline (19). Comparable phenomenon has been demonstrated in uphill running where step length (CL) was decreased and step frequency (CR) was increased with the elevated treadmill incline (39, 40). The adjustment of CL and CR in uphill running was coped with the uphill progression and the available metabolic power (40).

The relative poling time (Figures 3B, 4B) are greater at steeper incline with both technique in this study. Specifically, the time for getting ready for the next pole plant was shorter at steeper incline, and the proportion of cycle for generating pole forces increased with the elevated treadmill incline in both techniques (19). As the G3 technique contains both pole and ski thrusts, the ski movements were analyzed as well. The relative kicking time (Figure 4C) was independent from the incline of the treadmill. This indicated that in response to the increased treadmill incline, the proportion of cycle for generating ski forces would not change.

The relative overlap time (Figure 4D) at 4° and 5° were shorter when compared to that at 3°. The less relative overlap time indicated that the skier may start “seeking ground contact” with the new glide ski later at steeper grade (15). The magnitude of the relative overlap time in this study (23%–25%) was higher than that reported by Ohtonen et al. (15) (around 10%). This difference might be attributed to environmental difference (on snow vs. on treadmill) and athletes' level (a group of elite skiers vs. a group of diverse level skiers). Faster elite skiers may control balance more securely than averaged level skiers (15).

### 4.2. Forces and impulses

With both techniques, the PPF (Figure 3C and Figure 4E), and pole propulsive force impulse (Figure 3D and Figure 4F) increased continuously up to the steepest incline in this present study. Combined these results with results from cycle characteristics, although less time was used for getting ready for pole plant, greater pole force and pole propulsive force should be reached at steeper incline. These results were consistent with the previous study that in both DP and G2 technique, the force variables from poles were all greater at the steeper grade than the lower grade (19). 0.03–0.04 N*s ski propulsive force impulse was found (Figure 3F) for DP technique in this study. This result supports our hypothesis that with DP technique, we could measure some propulsive force from skis, but it would be quite small. The magnitude of the ski propulsive force impulse was very small and seems to be negligible. Therefore, from overcoming the total resistance point of view, DP technique could be considered as an upper-body movement as indicated by other previous studies (25–27).

Greater ski force and ski propulsive force should also be generated to overcome the increased total resistance at steeper incline with G3 technique, as the PKF and ski propulsive force impulse all increased at steeper incline in this study. However, although participants in this study had similar bodyweight with participants in other previous studies (15, 41), the magnitude of PKF in this study was lower than those in previous studies. The additional weight and height of the roller ski equipped with the force measurement bindings may decrease the usage of legs, thereby greater ski forces could not be reached. In addition, the difference in skiers' level and the skiing intensity level may also be attributed to the difference in PKF.

The gravity component parallel to the treadmill surface increased with the incline (38), thus more forces and greater power output are needed at a steeper grade. Therefore, the ACPF increased continuously with the elevated treadmill incline with both techniques (Figure 3G and Figure 5A). In response to the elevated treadmill incline, the power output in overcoming the total resistance increased by 45% and 50% with the DP and G3 technique respectively in this study (Figure 3H and Figure 5B). For DP technique, the propulsive impulse was mainly generated by poles and more pole propulsive force impulse are needed if skier intend to maintain the speed with increasing incline. But with G3 technique, increase the propulsive force generated from both poles and skis are needed. It is also worth remembering that in treadmill conditions skiers do not have to work against wind resistance as is the situation when skiing outside. Especially with higher speed, the wind resistance would have a great influence on propulsive forces (42). Therefore, the results about the magnitude of forces in this study may be different from studies which concentrated on snow skiing (15).

### 4.3. Effectiveness and contributions of ski and pole forces at different inclines

Effectiveness index has been used as a useful tool to evaluate athlete's overall economy on force production (16). The results of this study support our hypothesis that with DP technique, the effectiveness of pole force in overcoming the total resistance would be greater at steeper inclines than at relative lower inclines. The pole force effectiveness increased by 7% from 3° to 5° with DP technique, indicating that a greater proportion of the resultant force is generated to overcome the total resistance and a higher overall economy on force production. For DP technique, the increase in power output in overcoming the resistance was mainly due to the increase in pole force effectiveness because none of the propulsive force could be obtained by skis.

The results of our study support our hypothesis that pole forces would contribute more to overcome the total resistance and more effective than ski forces at any incline. Our results indicated that the relative contributions of pole forces were 5–6 times greater than the relative contributions of ski forces (Figure 5D), and 55%–58% of the resultant pole forces is generated to overcome the external resistance which is greater than the effectiveness of ski force (0.9%–1.6%, Figure 5C). A previous study demonstrated that for G3 skating technique, about two thirds of propulsive is due to the pole forces and one-third due to the ski forces (33). The difference between our current study and the previous study (33) may be attributed to the difference in treadmill incline and speed. In addition, the extra weight and height of roller-skis caused by the force measurement bindings may decrease the usage of legs. Therefore, more pole force than ski forces were used in overcoming the total resistance. However, the results of our study do not support our hypothesis that the relative contribution of ski forces to overcome the total resistance would increase at steeper grade. Although the relative contribution from ski and pole forces were affected by the incline of the treadmill, the effects were medium ($\eta_{p}^{2}$ = 0.106), and the magnitude of the change did not vary so much. Specifically, increasing the propulsive force generated from both poles and skis are needed at steeper grade but the contribution ratio will not change.

However, what should be noted was that the contribution mentioned in this study were the relative amount of force to overcome the external resistance. The internal work, which is used to move the internal structures and not used to perform work on external objects (43), was not included. A previous study reported that about 37%–46% of the external power was contributed by the trunk and legs in DP technique (38). During the recovery phase of the DP technique or the pure gliding phase in the V2 skating technique, the lower extremity also contributes to repositioning the body, which may help skiers enhance the use of body weight (29, 44) and may increase the forces generated from skis and poles. However, this kind of contribution of lower extremity could not be revealed by the propulsive force. Therefore, only 0.9%–1.6% of the ski forces were generated to overcome the resistance. Results from this study suggests that the role of legs is quite small, but this might be affected by the height and weight of the roller ski and the level of study group. This result needs to be confirmed with higher level athletes and more advanced measurement equipment.

Our study has several limitations. The first limitation is that subjects in this study had varying skiing levels, and we recruited make subjects only. Therefore, future studies with a group of more skilled skiers of both genders will enhance the generality of our conclusion. The measurements of this study were performed in an indoor laboratory and on the treadmill. The lack of wind resistance (42) and the motor and belt of the treadmill (45) may prevent the results of this study from being directly applicable to snow skiing. In addition, the roller skiing equipment used in this study contained force measurement sensors, which are heavier and add extra height compared to the normal ones, may affect the skiing techniques. Future study could reduce the impact of measurement equipment by using portable force measurement roller skis (46) and lighter force measurement poles to help the results more easily transferable to daily roller ski training.

## 5. Conclusions

The present study provides detailed biomechanical information of DP and G3 techniques at three different uphill inclines. The higher power output in overcoming the total resistance was required to manage skiing at a greater incline. With DP technique this was supplied by greater pole forces and pole force effectiveness, which means that the upper body demands, and technical effectiveness were increasing with incline. This fact plays a role when skiers using DP also to a greater extent in moderate uphill sections like e.g., in “Visma Ski Classic” race events or when skiers are forced to use DP in uphill sections due to worse grip wax conditions for diagonal skiing. With G3 technique, increasing both pole and ski force effectiveness were needed at steeper grade, but the much larger relative contribution of pole forces vs. ski forces in overcoming the total resistance did not change over incline. This underlines the crucial role of external pole work for propulsion during G3 over different terrains while the role of legs may stay more in supporting the body against gravity and repositioning body segments.

## Data Availability

The original contributions presented in the study are included in the article/Supplementary materials, further inquiries can be directed to the corresponding author.
